# Aberrant epigenetic regulators control expansion of human CD34+ hematopoietic stem/progenitor cells

**DOI:** 10.3389/fgene.2013.00254

**Published:** 2013-11-28

**Authors:** Farnaz Faridi, Kanagaraju Ponnusamy, Isabell Quagliano-Lo Coco, Linping Chen-Wichmann, Manuel Grez, Reinhard Henschler, Christian Wichmann

**Affiliations:** ^1^Department of Transfusion Medicine, Cell Therapeutics and Hemostasis, Ludwig-Maximilian University HospitalMunich, Germany; ^2^Institute of Transfusion Medicine and Immunohematology, Goethe UniversityFrankfurt, Germany; ^3^Institute for Biomedical ResearchGeorg-Speyer-Haus, Frankfurt, Germany

**Keywords:** epigenetics, leukemia, stem/progenitor cell expansion, HSPC, RUNX1/ETO

## Abstract

Transcription is a tightly regulated process ensuring the proper expression of numerous genes regulating all aspects of cellular behavior. Transcription factors regulate multiple genes including other transcription factors that together control a highly complex gene network. The transcriptional machinery can be “hijacked” by oncogenic transcription factors, thereby leading to malignant cell transformation. Oncogenic transcription factors manipulate a variety of epigenetic control mechanisms to fulfill gene regulatory and cell transforming functions. These factors assemble epigenetic regulators at target gene promoter sequences, thereby disturbing physiological gene expression patterns. Retroviral vector technology and the availability of “healthy” human hematopoietic CD34+ progenitor cells enable the generation of pre-leukemic cell models for the analysis of aberrant human hematopoietic progenitor cell expansion mediated by leukemogenic transcription factors. This review summarizes recent findings regarding the mechanism by which leukemogenic gene products control human hematopoietic CD34+ progenitor cell expansion by disrupting the normal epigenetic program.

## Purification and genetic manipulation of human hematopoietic CD34+ progenitor cells

**Figure 1:**
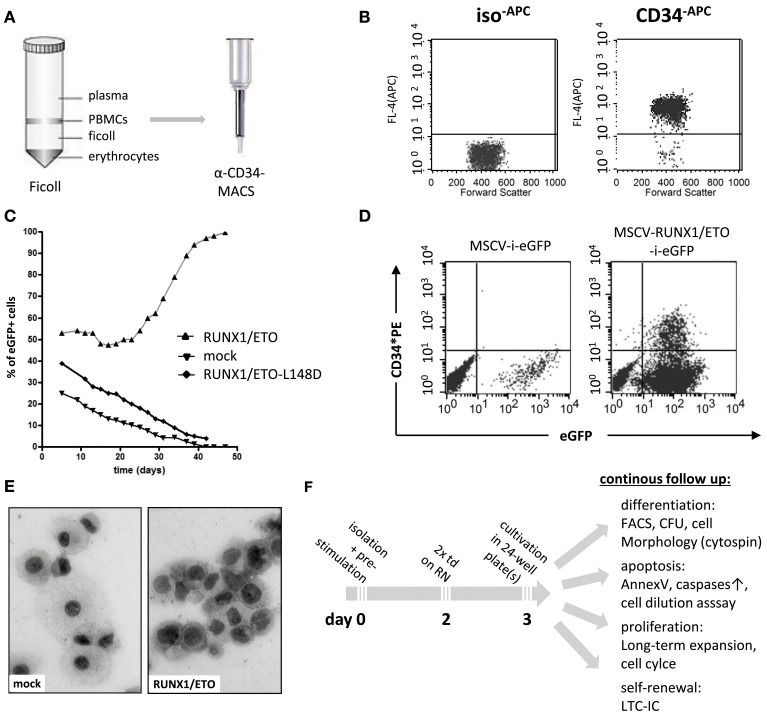
**Purification and genetic manipulation of human hematopoietic CD34+ progenitor cells. (A)** Isolation of human CD34+ progenitor cells using a Ficoll gradient procedure and MACS column purification following labeling with anti-CD34 microbeads. **(B)** FACS analysis after MACS showing purified CD34+ cells. **(C)** Oncogene-mediated selection of RUNX1/ETO-expressing human progenitor cells in *ex vivo* culture after retroviral transduction with the indicated vectors co-expressing eGFP. RUNX1/ETO-L148D, DNA-binding defective mutant. **(D)** FACS analysis of CD34 surface marker expression of mock- and RUNX1/ETO-transduced human progenitor cells at week 4. **(E)** Cytomorphological analysis of mock- vs. RUNX1/ETO-expressing cells following the *ex vivo* selection process. **(F)** Experimental design of CD34+ cell long-term expansion analyses. PBMC, peripheral blood mononuclear cell; MACS, magnetic cell separation; MSCV, murine stem cell virus; i, IRES, internal ribosome entry side element; eGFP, enhanced green fluorescent protein; RN, retronectin; CFU, colony forming unit; LTC-ICs, long-term culture-initiating cells; td, transduction.

Human hematopoietic progenitor and stem cells (HPSCs) are characterized by high expression levels of the cell surface antigens CD34 and CD133 and the lack of CD38 expression for more primitive cells. CD34 is a member of the single-pass transmembrane sialomucin protein family and a cell-cell adhesion factor expressed in some cell types, including hematopoietic cells, endothelial cells, muscle satellite, hair follicle stem cells, and fibrocytes. The CD34 antigen has been widely used as a cell surface marker to identify and isolate HPSCs from bone marrow or peripheral blood. However, the exact biological function of CD34 remains largely unknown. Recent reports have demonstrated that CD34 can alter the cell adhesion, migration, and engraftment potential of hematopoietic progenitor cells in the bone marrow niche (Fackler et al., 1995; Healy et al., 1995; Cheng et al., 1996; Nielsen et al., 2007; Salati et al., 2008). For experimental purposes, human CD34+ blood progenitor cells can be obtained as a residual product following bone marrow aspiration or blood cell apheresis subsequent to stimulation and mobilization of bone marrow CD34+ cell precursors with granulocyte colony stimulating factor (G-CSF). Moreover, several companies provide human CD34+ purified HPSCs that test negative for infectivity complete with an ethical agreement for experimental usage. Placenta-derived cord blood cells may also be applied for long-term expansion experiments, as these cells display stemness and long-term engraftment potential superior to that of adult CD34+ cells (Hao et al., 1995). Recently, it has been shown that placenta-derived amniotic fluid stem cells expressing the c-KIT cell surface marker represent a new and attractive source of primitive hematopoietic progenitors for experimental purposes (Perin et al., 2008). Currently, two major techniques used to enrich CD34+ cells are available, fluorescence-activated cell sorting (FACS) and magnetic activated cell sorting (MACS). Although FACS enables the purification of CD34+ cells, the antibody labeling and sorting procedures act as significant cellular stress stimuli. MACS represents another rapid and efficient enrichment procedure, which has become the current standard and most convenient method used to purify CD34+ cells (Clarke and Davies, 2001). Magnetic labeled CD34 antibodies are used to recover high-purity populations of CD34+ cells by passing cells through magnetic columns (Figures 1A,B).

However, it is a challenge to achieve long-term expansion of CD34+ progenitor cells while maintaining their immature state *ex vivo*, even in the presence of highly concentrated cytokines and supportive adherent cells of mesenchymal origin (Zhang et al., 2008; Weisel et al., 2009). To establish *ex vivo* long-term expansion using an alternative approach, leukemia-associated oncogenes can be delivered and stably expressed by the retroviral gene transfer technology. The most commonly used delivery system is the gamma-retroviral vector system based on the Moloney murine leukemia virus (Mo-MLV) genome (Kohn et al., 1987). The murine stem cell virus (MSCV) expression vector is one of the most frequently employed gamma-retroviral vector systems, as it enables stable and high transgene expression in virtually all cell types (Hawley et al., 1994). Lentiviral vectors, which are based on the HIV genome, display an increased capacity to incorporate large transgenes (up to 10 kilobases); although, vector titers decrease when using larger inserts (Matrai et al., 2010). Lentiviral transduction efficacy can be further improved by concentrating the viral particles via ultracentrifugation (Naldini et al., 1996; Kanbe and Zhang, 2004). RetroNectin-based gene transduction protocols dramatically enhance the efficiency of retrovirus-mediated gene transfer in hematopoietic suspension cells. With this system, retroviral particles are preloaded onto RetroNectin-coated surfaces and co-localize viral particles and target cells into close proximity, thereby markedly increasing the transduction efficiency (Hanenberg et al., 1996). Expression of a gene of interest is usually coupled to the expression of a marker gene, e.g., enhanced green fluorescent protein (eGFP), which allows for the immediate determination of viral transduction efficacy and the identification of transduced cells to assess proliferation, differentiation and cell death (Figures 1C–F).

## Leukemic transcription factors epigenetically control progenitor cell expansion

In principle, the term epigenetic regulation refers to any stable mitotically perpetuated regulatory mechanism of a genome that does not alter the primary nucleotide sequence (Jaenisch and Bird, 2003; Oki and Issa, 2010). DNA methylation, histone modification, histone variant deposition in gene bodies and recruitment of transcription-related enzymes to specific genetic loci are the most commonly known molecular mechanisms that mediate epigenetic phenomena. DNA methyltransferases (DNMTs) are the key enzymes of genome methylation, which play an important role in the epigenetic regulation of gene expression and repression (Jackson-Grusby et al., 2001; Jaenisch and Bird, 2003). In general, DNMT1 maintains DNA methylation in mammalian cells, while DNMT3A and DNMT3B act as *de novo* DNMTs by methylating unmethylated CpG sites (Oki and Issa, 2010). Recent studies have demonstrated that DNA methylation is critical for the self-renewal and differentiation of normal and leukemic stem cells (Hogart et al., 2012). Moreover, posttranslational modification of histones regulates chromatin structure and transcription. Histone acetylation and methylation alter gene expression patterns and cellular behavior during the onset and progression of oncogenesis (Ellis et al., 2009). Furthermore, repressive histone modification mediated by Polycomb-group (PcG) complexes is involved in the balance between the self-renewal and differentiation of hematopoietic stem cells via regulation of the cell cycle. PcG proteins are histone modifiers found in two protein complexes, Polycomb Repressive Complex (PRC) 1 and PRC2, which target cis-regulatory polycomb response elements (PREs) by normal and aberrant transcription factors (Cedar and Bergman, 2009). PRC2, the “initiating complex”, catalyzes the di- and tri-methylation of histone H3 at lysine 27 (H3K27me3) accompanied by the gene repression and maintenance of self-renewal programs of leukemic stem cells (Sashida and Iwama, 2012). Following PRC2-mediated histone methylation, the PRC1 complex (“maintenacnce complex”) is recruited to chromatin via binding to H3K27me3. Forced expression of PcG genes, such as BMI1 or EZH2, enhances the self-renewal capacity of HSCs and obviates long-term repopulating exhaustion during serial transplantation (Iwama et al., 2004). In the following paragraphs, we describe the mechanism by which aberrant transcription factors deregulate gene expression, thereby promoting hematopoietic CD34+ progenitor cell expansion by forming aberrant epigenetic regulator complexes that perturb gene expression.

### RUNX1/ETO assembles multiple epigenetic regulators

**Figure 2:**
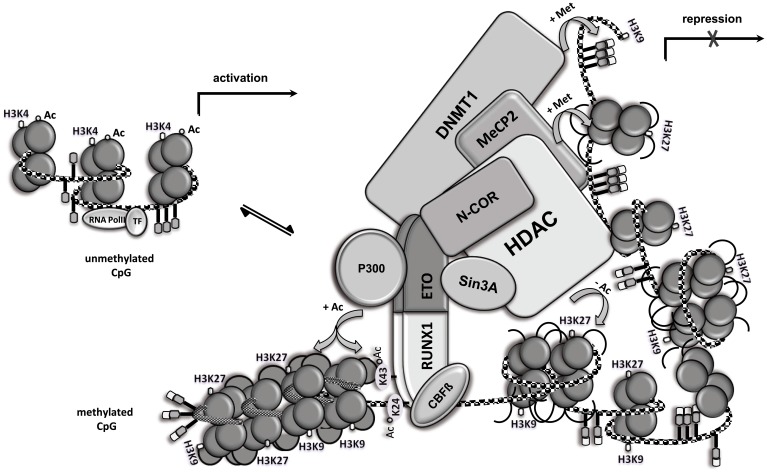
**RUNX1/ETO assembles multiple epigenetic regulators.** Open chromatin (left side) and chromatin modification following RUNX1/ETO DNA-binding and high molecular weight complex formation. RUNX1/ETO recruits several proteins, including N-CoR, mSIN3A, HDAC, MeCP2 and DNMT1, into a high molecular weight complex that triggers chromatin condensation (right side), thereby repressing gene expression. Locus-dependent, RUNX1/ETO recruits the co-activator p300 and mediates acetylation of RUNX1 at the two conserved lysine residues and nearby histones, thereby promoting gene transcription. Ac, acetylation; Met, methylation; CpG, CpG site; TF, basal transcription factor.

The transcription factor RUNX1 is one of the most frequent genes involved in chromosomal translocations found in acute myeloid leukemia (AML). RUNX1 is the sequence-specific DNA-binding subunit of the core binding factor and a key regulator of normal hematopoiesis (Zaiman et al., 1995). RUNX1 activity can be altered by various genetic and epigenetic events, including mutations, deletions, and chromosomal translocations. The translocation *t*_(8, 21)_ generates the RUNX1/ETO fusion protein, which controls a highly complex gene network of several thousands of genes via its RUNX1 DNA-binding domain (Martens et al., 2012). The ETO portion contains four highly conserved nervy homology region (NHR) domains, which together assemble several transcriptionally active regulators. The ETO NHR2 and NHR4 domains are important for interaction with co-repressor molecules such as mSIN3A, N-CoR and SMRT, which recruit histone deacetylases (HDACs) 1–3, thereby contributing to heterochromatin formation. RUNX1/ETO deletion mutants lacking the NHR2 and NHR4 domains are completely defective in their capacity to induce human CD34+ progenitor cell expansion (*own observation*). RUNX1/ETO further directly interacts with DNMT1, the major DNA methylating enzyme of a complex containing N-CoR and mSIN3A. Recruitment of DNMT1 to the interleukin-3 promoter causes gene repression, which can be reverted via administration of demethylating agents (Liu et al., 2005). The RUNX1/ETO high molecular weight complex also contains the methyl-CpG-binding protein 2 (MeCP2), which enhances the silencing of target genes that regulate the proliferation and differentiation of hematopoietic cells (Fazi et al., 2007). However, the central NHR1 domain provides a docking site for the transcriptional activator p300, serving to activate genes involved in self-renewal promotion such as ID1 and p21 (Wang et al., 2011). P300 mediates acetylation of RUNX1 itself at two conserved lysine residues within the DNA-binding domain and may acetylate histone tails to open chromatin. Hence, pharmacological inhibition of p300 induces the cell growth arrest of RUNX1/ETO-expanded human CD34+ cells by selectively blocking its transcriptional activation function (Yamaguchi et al., 2004; Wang et al., 2011). These reports suggest a model in which RUNX1/ETO either epigenetically activates or represses target genes, depending on the concurrent interaction with both repressive elements and p300 (Figure 2). The exact fine-tuning of specific cofactor binding may be dependent on the genomic locus and the posttranslational modification of the RUNX1/ETO cofactor recruitment domains. Importantly, the singular overexpression of RUNX1/ETO has been shown to induce robust *ex vivo* expansion of “healthy”, human hematopoietic CD34+ progenitor cells. The RUNX1/ETO-selected progenitor cells continue to express the CD34 antigen and are able to self-renew. The cells can be cultured for more than 7 months while retaining telomerase activity but remain cytokine dependent for survival and proliferation. However, in contrast to primary AML patient samples, RUNX1/ETO-selected progenitor cells fail to induce leukemia development in immunocompromised NOD/SCID mice, thereby suggesting that additional genetic events are necessary for leukemia development (Mulloy et al., 2002, 2003). This *ex vivo* cell expansion model was also applied to analyze second hit mutations able to enhance the *ex vivo* expansion capacity of the epigenetic regulator RUNX1/ETO. Retrovirally co-expressed N-RAS(G12D), a frequent second hit mutation associated with *t*_(8, 21)_ AML, has been shown to increase CD34 expression levels and colony formation numbers, enhance replating capacity, rendered cells cytokine independent in growth and improved cell engraftment in NOD/SCID mice, thereby suggesting that N-RAS plays a critical role in the stepwise transformation of *t*_(8, 21)_ leukemia (Chou et al., 2011). However, the exact mechanism by which RAS signaling complements the epigenetic changes induced by RUNX1/ETO remains unclear.

### MLL fusions are potent inducers of CD34+ progenitor cell expansion

The *MLL* (mixed lineage leukemia) gene is a human homologue of the *Drosophila melanogaster* trithorax gene and a recurrent target of chromosomal translocations found in acute leukemia. MLL encodes a large multi-domain DNA-binding protein that confers transcriptional activation and repression via histone modifying methyltransferase activity. Histone H3 lysine 4 (H3K4) methylation of target gene promoter regions ensures proper function of the RNA polymerase II. MLL mediated trimethylation of histone H3 lysine residue 4 (H3K4me3) is a critical step involved in the epigenetic maintenance of gene transcription in hematopoietic stem and progenitor cells (Follows et al., 2003; Krivtsov and Armstrong, 2007). Malignancies due to MLL translocations that give rise to chimeric proteins with more than 70 known fusion partners confer a generally poor prognosis. MLL/AF9, MLL/ENL, and MLL/AF4 fusions represent the most prevalent MLL fusion protein forms. Various studies have suggested that MLL fusion partners are generally involved in chromatin remodeling (Krivtsov and Armstrong, 2007). MLL fusion proteins upregulate HOX genes, which are important regulators of hematopoietic cell maturation. Consecutively proliferative hematopoietic progenitor cells rapidly expand and display a block in cellular differentiation. Aberrant expression of HOXA9, for example, induces a myeloid differentiation block via co-recruitment to promoter regions bound by multiple myeloid transcription factors including PU.1, RUNX1, and C/EBPα (Mereau and Schwaller, 2013). All fusion proteins share the N-terminus of the MLL histone methyltransferase protein, but lack the C-terminal SET (Su(var)3–9, enchancer-of-zeste, trithorax) domain with intrinsic H3K4 methyltransferase activity (Slany, 2010). Three related protein complexes directly interact with MLL fusion proteins. The polymerase-associated factor complex (PAFc) regulates RNA polymerase II activity. The positive transcription elongation factor b (P-TEFb) and histone methyltransferase DOT1L are then recruited to MLL target gene promoter sites. P-TEFb phosphorylates the C-terminal repeat domain of RNA polymerase II, thereby stimulating transcriptional elongation. By methylating histone H3 at lysine 79, recruited DOT1L also positively regulates transcriptional elongation, a critical step for maintaining MLL fusion protein-induced leukemogenesis (Slany, 2010; Bernt et al., 2011; Ballabio and Milne, 2012). DNA binding is achieved via the MLL N-terminus, the MLL complex partner MENIN, and LEDGF. Several studies have shown that MLL-rearranged leukemia cells treated with hypomethylating agents regain expression of tumor suppressor genes, thereby leading to cell growth inhibition. Histone deacetylation via direct recruitment of HDACs to wild type MLL-bound promoters may further function to promote MLL fusion protein-triggered leukemic cell expansion and leukemogenesis (Bernt and Armstrong, 2011). MLL translocations markedly deregulate epigenetic control mechanisms and may therefore require no or only few additional mutations to cause leukemia in humans (Armstrong et al., 2002; Neff and Armstrong, 2013). In contrast to AML gene rearrangements, ectopic expression of either MLL/AF9 or MLL/ENL alone confers human CD34+ cell expansion and results in myeloid or lymphoid leukemia development in a NOD/SCID transplantation mouse model (Barabe et al., 2007; Wei et al., 2008). Cell morphological analysis has revealed that AML, B-ALL, and mixed lineage leukemias are associated with both MLL rearrangements. Remarkably, even after 70 days of *ex vivo* culture, the expanded progenitor cells gave rise to leukemia development when transplanted into NOD/SCID mice. In contrast, MLL/AF4, which also enhances CD34+ progenitor cell expansion, did not induce leukemia even after intra-bone marrow transplantation into NOD/SCID mice (Montes et al., 2011). The underlying reason for the striking differences between individual MLL fusion genes in their ability to induce leukemia onset in NOD/SCID mice remains almost completely unknown. A direct comparison of deregulated genes may reveal which additional pathways are activated in MLL/AF9 and MLL/ENL leukemic human CD34+ cells.

### BMI1 is essential for normal and leukemic CD34+ cell expansion

BMI1 (B-cell specific Moloney insertion site-1) is a transcriptional repressor belonging to the PcG gene family. BMI1 acts as a chromatin regulator and is a central core component of the PRC1 protein complex that binds at genomic loci marked by both repressive H3K27me3 and activating H3K4me3 methylation modifications. The PRC1 complex has no intrinsic methyltransferase activity, however, BMI1 directly interacts with the DNMT-associated protein 1 (DMAP1) to ensure the methyltransferase activity of DNMT1 and the proper assembly of the PRC1 complex. Recognition of PRC2 methylated histone H3K27 promotes the recruitment of the PRC1 complex. This complex subsequently acts as a recruitment factor for E3 ligase, which is necessary for the efficient ubiquitination of H2A-K119, thereby promoting gene repression by inhibiting RNA polymerase II-dependent transcriptional elongation and inducing chromatin condensation (Ohtsubo et al., 2008; Schuringa and Vellenga, 2010; Nacerddine et al., 2012). Transcriptional silencing of the *INK4a* locus plays a crucial role in the maintenance of HSC self-renewal capacity regulated by BMI1, which simultaneously mediates the repression of the *p16*^*INK*4*a*^ and *p19*^*ARF*^ genes (Sashida and Iwama, 2012). BMI1 further interferes with central tumor suppressor pathways associated with retinoblastoma protein and p53. P53 specifically binds to the *ARF* locus and recruits HDACs. Deacetylation facilitates the recruitment of PRC to the *ARF* locus, thereby promoting H3K27 trimethylation and the silencing of ARF expression (Zeng et al., 2011). Expression of BMI1, which is also tightly regulated, declines once hematopoietic progenitors begin to differentiate (Lessard et al., 1999). Forced downregulation of BMI1 in human normal CD34+ cells and acute leukemic CD34+ cells impairs their long-term expansion and self-renewal capacity (Rizo et al., 2009; Harada et al., 2013). In contrast, forced expression of BMI1 enhances the symmetrical cell division of HSCs, their engraftment potential and the *ex vivo* CD34+ cell expansion capacity (Iwama et al., 2004; Schuringa and Vellenga, 2010; Nakamura et al., 2012). It has also been suggested that BMI1 overexpression confers protection against senescence and apoptosis by recruiting into sites of DNA damage and histone ubiquitination (Nakamura et al., 2012). Overexpression of BMI1 also enhances the leukemogenic activity of BCR/ABL, a fusion protein recurrently found in chronic myeloid leukemia (CML). Co-expression of BMI1 and BCR/ABL has been shown to trigger *ex vivo* expansion of human CD34+ progenitor cells for more than 20 weeks in culture and promote leukemic transformation of healthy human CD34+ cells when transplanted into NOD/SCID mice (Rizo et al., 2010). Similarly, overexpressed BMI1 transforms and reprograms CML B-lymphoid progenitors into leukemia-initiating and self-renewing stem cells, thereby causing B-cell acute lymphoid leukemia *in vivo* (Sengupta et al., 2012). Moreover, BMI1 overexpression is frequently observed in myelodysplastic syndrome (MDS) patients harboring RUNX1 mutations. Accordingly, a recent report has shown that BMI1 overexpression enhances the proliferation capacity of mutated RUNX1 pre-transformed human CD34+ cells. A stepwise transduction of mutated RUNX1 followed by BMI1 overexpression in human CD34+ cells results in long-term CD34+ progenitor cell proliferation with a retained CD34+ cell fraction similar to the phenotype observed in patients with higher risk MDS (Harada et al., 2013).

## Synopsis

The availability of “healthy” human hematopoietic CD34+ progenitor cells and retroviral vector technology facilitates the establishment of models used to analyze human leukemic CD34+ progenitor cell expansion driven by leukemia-associated gene products. Although this approach may hardly be suitable as a cell therapeutical method for progenitor cell production due to the danger associated with stable integrated oncogenes, the herein described *ex vivo* cell expansion system allows for the precise analysis of the molecular determinants of aberrant epigenetic regulation that control CD34+ cell expansion. Moreover, this cellular readout system can further be utilized to test inhibitors targeting leukemic gene products and their epigenetic machinery as well as to screen complex drug libraries to identify oncogene inhibitors. Promising candidates can be developed further as lead compounds in preclinical cellular transformation assays and mouse leukemia models.

### Conflict of interest statement

The authors declare that the research was conducted in the absence of any commercial or financial relationships that could be construed as a potential conflict of interest.
